# Case of Aortitis, with Autopsy, and Remarks

**Published:** 1845-06-01

**Authors:** Geo. N. Burwell


### Case of Aortitis, with Autopsy, and Remarks:

Communicated for the Buffalo Medical Journal, by GEO. N. BURWELL, M. D.

---

My Dear Doctor—

I send you an interesting case of Aortitis or more properly perhaps, Arteritis. The patient came under my care, while attached as Resident Physician, to the Philadelphia Hospital, Blockley, and offered the only known case of this form of inflammation, during the year of my residence in that institution.

Yours, very truly, GEO. N. BURWELL.

---

Peter Stevens, black, aged sixty-three years, was brought to the Hospital, late in the afternoon of February 13th, 1843. He was carried to his ward, not being able to walk. The physician who received him, said that he complained of intense pain in his chest, without being able to localize it; "it was all through it," to use his own expression. He felt his pulse, and found it nearly natural, both in frequency and strength. He had no fever. These observations were hurriedly made, however, and ought probably, to be received with some degree of allowance.

I first saw him about 8 o'clock in the evening; he was lying in his bed on his left side, his head bent forward, with his chin nearly touching his chest, gasping for breath, his lower jaw falling at every inspiration; at times his eyes would roll up, which, with the anxious yet relaxed state of his countenance, gave him a deathly appearance. His intelligence was perfectly good. He answered our questions with difficulty, and at times, they had to be repeated. We found that about three weeks before he was taken with symtoms of Pleurisy of the left side, from which he had never entirely recovered; but had kept at his work as a common porter, and had been thus employed all the day previous. At one o'clock in the morning of the 13th, he had been thus attacked suddenly, and while in bed. The pain in his chest, above spoken of constituted his greatest misery. An order for admittance to the Hospital, was obtained for him during the forenoon, and he was brought in in the afternoon without having received any medical assistance.

A bowel complaint in the meantime had come on, which obliged him to get up to stool every fifteen or twenty minutes, the discharge being thin, and passed with much straining. He found the position on the left side with his head low, the most comfortable one, although, from his difficulty of breathing, it would have been supposed he would have been easiest sitting up. He could not lie on his back without aggravation of his sufferings. The nurse informed me that from the time of his entrance, he complained of great distress across the upper portion of the sternum. Not much was said to him, as he disliked being talked to. On examining his chest, as well as I could without adding to his pain, I found on the left side almost total flatness on percussion, from the second rib to the base of the chest, and to the left axilla; the flatness did not extend much to the right of the sternum. Percussion of the right side of the chest, and over the left lung, posteriorly, resonant. The respiratory murmur could be heard clear at the apex of the left lung, and throughout the right lung. No murmur heard over the lower lobe, posteriorly, where the percussion was clear; between the second and fourth ribs of the left side anteriorly, there was bronchial respiration, although not very loud.

The sounds of the heart could no where be heard, nor could its impulsion be felt. His pulse was scarcely perceptible, it was so small and feeble; it could not be felt in the left wrist when he lay upon his left side. Its frequency was about 110 beats in the minute.

No diagnosis was made out. The symptoms of any of the common diseases of the chest were not well enough marked, to found upon them a definite opinion. He died in two or three hours after I left him.

*Post-mortem, thirty-eight hours after death.* The body was that of a large powerful negro, full six feet and two to four inches high, and not at all emaciated; some froth on the lips; blood ran from the nose on turning the body over; face somewhat bloated. Pleuritic adhesions of pleuræ of both lungs, which were of some standing, yet easily broken; lungs congested, but crepitant. Heart hypertrophied and dilated, but the relative size of the cavities not lost; slight thickening of the aortic valves. On cutting into the aorta, its internal coat was found to be of a bright scarlet color, which extended throughout the whole length of the vessel, and down the femoral arteries until they became popliteal ; the injection, also, extended into the carotid, subclavian, and coeliac arteries. The other arteries not examined. A piece cut from the middle portion of the humeral artery, did not exhibit this redness. The arteries which were inflamed, were empty, except a small clot near the aortic valves. The endocardium was not affected ; dark, slightly coagulated clots occupied the cavities of the heart, and extended into the pulmonary artery, the inner coat of which was also reddened, but not near to the extent of that of the aorta, and of a duller color. The inflamed membrane was not thickened nor was any fibrous effusion noticed. In the cellular tissue under it, were noticed small spots or flakes, of what appeared to be ætheromatous deposit, which was found quite thickly deposited in the entire length of the aorta. The venæ cavæ had blood in them, and the lining membrane was very lightly tinged of a dark reddish brown color, evidently the result of imbibition merely. There was an ounce of dark bloody fluid in the pericardium and about the same quantity in the left pleuræ. The mucous membrane of the stomach was slightly inflamed in two or three places. Other organs of abdomen healthy. Brain not examined.

On reading the above, the first question that will arise, will, probably, be whether this was really a case of inflammation, or merely one of cadaveric strain. A great deal has been written on this point, and in deciding upon the true pathology of the case, it is an important one. There is no doubt in my mind as to the inflammatory nature of the redness. The marked difference between the bright color of the aorta, and the sombre stain of the pulmonary artery, the still fainter color of the venæ-cavæ, and the healthy state of the endocardium, all of which had either fluid or clotted blood in contact with their lining membrane, demonstrate this point conclusively. There are only two facts which afford the least reason to doubt this, viz : the fluid state of the blood, and the length of time which intervened between death and the post-mortem examination.

The case is interesting from its being one of the most rapidly fatal cases of arteritis on record. A strong man, healthy, with the exception of some pain in his side for two or three weeks, which was not severe enough to confine him to the house, after an ordinary day's work, is awakened suddenly in the night with severe pain all through his chest, which lasts without intermission, till his death, about twenty-two hours from the first accession of pain. Did the inflammation exist before the pain was felt ? I know no way of determining this ; probably not, in its acute form, at least. What connection was there between the inflammation and the little, opaque, cream-colored deposits, noticed so thickly beneath the inner membrane ? Their consistence would indicate in them a greater age than the inflammation had, but it does not follow that, of course, they were the exciting cause of the inflammation. This point must remain undecided.

The next question that arises, is, what is the connexion between the inflammation and the agonizing pain suffered by the patient ? Was it angina-pectoris? If so, the case would go far in support of Dr. Corrigan's opinion, that "inflammation of the lining membrane of the mouth of the aorta, is capable of producing the group of symptoms to which we give the name of Angina-pectoris, and is, therefore, entitled to a place in the list of the causes of that affection."* It does not appear in the notes that he had pain in the left arm, and I do not recollect of asking him the question. If I did not, he would not have complained of it, for such was his distress, that he complained to me of nothing, and appeared as though he wished to be left alone to die. Besides, he did not die the sudden death of those who die of angina-pectoris.

The case is of high interest in a pathological point of view, and perhaps also, in a legal sense. Therapeutically considered, it is not of much value, at least, after I saw him, for he was then dying, beyond the reach of medicine.

Before closing this article, it may be well to take a glance at the varieties of acute arteritis. There appears to be three forms of it. The *first*, and most common variety, is that occurring with Endo carditis, where the inflammation extends into the aorta, more or less. This cannot well be distinguished from the Endo-carditis, nor is it important that it should be. It is, merely, an aggravated and extended form of this inflammation, and as such, renders the prognosis more grave without materially altering the treatment. This variety, also, sometimes complicates cases of Pericarditis, and of Pleuro-pneumonia.

The *second* variety, is seated in the extremities, and far more frequently attacks the legs, than the arms. It may be primary, or secondary ; as when it supervenes upon amputation, or upon a diseased state of the coats of the arteries of the limbs. Reported cases of it may be found scattered through the different medical journals. Dupuytren, in his Surgical Lectures, gives two cases. It has often, probably, been confounded with phlebitis, but may be distinguished from it by two signs ; 1st, the reduction, in arteritis, of the temperature of the limb, and 2d, by the occurrence of gangrene. Arteritis would seem to lead to gangrene, while phlebitis gives rise to œdema. This variety, having commenced in the limbs, is not always confined to them, but may travel, gradually, to the aorta, and on to the heart.

The *third*, and rarest form of the disease, is seen in those cases where the inflammation first attacks the aorta, extends to its branches, and perhaps also, to the endo-cardium. The case I have given belongs to this class. It is not, as yet, susceptible of positive diagnosis, but where the patient survives a number of days, it might be guessed at by the negative evidence of exclusion, with more or less certainty, according to the skill, tact and experience of the physician.

*Buffalo*, *May* 10th, 1845.

---

*For an abstract of Dr. Corrigan's paper, see Am. Med. Jour. old series, vol. 22. p467.

